# Impact of horticulture therapy on sleep quality in geriatrics

**DOI:** 10.6026/973206300213518

**Published:** 2025-10-31

**Authors:** Navaneetha Krishnan A, Siva Subramanian N, Mahalakshmi B

**Affiliations:** 1 Department of Psychiatric Nursing, Nootan College of Nursing, Sankalchand Patel University, Visnagar, Gujarat, India; 2 Department of Paediatric Nursing, Nootan College of Nursing, Sankalchand Patel University, Visnagar, Gujarat, India

**Keywords:** Horticulture therapy, sleep quality, geriatrics, non-pharmacological intervention, elderly care

## Abstract

Sleep problems are common in the elderly, affecting overall health and well-being. A pre-experimental one-group pre-test post-test study was conducted among 50 elderly in geriatric homes of Gujarat, where participants received six weeks of horticulture therapy. Sleep quality was measured using the Pittsburgh Sleep Quality Index. Data showed significant improvement, with severe sleep problems reducing from 92% to 0% and most participants shifting to mild or moderate categories (t = 27.768, p < 0.001). Thus, we show that horticulture therapy is an effective, safe and low-cost non-pharmacological intervention to enhance sleep quality in older adults.

## Background:

As individuals age, sleep quality often diminishes due to physiological changes, environmental factors and increased prevalence of health issues, contributing to impaired physical and psychological well-being [1]. Safe, non-pharmacological interventions are especially valuable in geriatric care settings, where the elderly are particularly vulnerable to polypharmacy and its side effects. One promising approach is horticultural therapy (HT)-a structured, plant-based intervention-increasingly recognized for its holistic benefits across mental, physical and social domains [2]. HT broadly encompasses activities like planting, nurturing and observing greenery, designed to stimulate sensory engagement and promote emotional restoration. It has demonstrated positive effects such as reduced stress, improved mood, increased self-esteem and enhanced social interaction, all of which may be intertwined with improved sleep [3]. Research involving residents of long-term care facilities observed enhanced physical agility, psychological well-being and a holistic sense of purpose following engagement in HT programs [4]. Even minimal indoor gardening tasks-like watering plants for just 10 minutes-have shown measurable stress reduction in older adults [5]. Sleep improvement among older adults via nature-based therapies has also been documented [6]. In a study in Singapore, participants in a therapeutic horticulture program maintained healthy sleep patterns, showed reduced anxiety, improved cognitive functioning and reported heightened happiness [7]. Similarly, a six-week HT intervention in Taiwan involving elderly participants from residential facilities and day-care centers led to significant improvements in sleep quality, alongside enhanced happiness, life satisfaction and strengthened immunity markers [8]. These findings suggest a compelling biological and psychological basis for improved sleep outcomes: sensory engagement in nature, reduced stress, improved mood, physical activity and social connection each appear to contribute to restoring more restful sleep in the elderly [9]. Despite this promising evidence, research specifically targeting sleep quality in geriatric home settings remains limited. Therefore, it is of interest to describe the impact of horticulture therapy on sleep quality among elderly residents of geriatric homes.

## Methodology:

## Research design:

The present study adopted a pre-experimental one-group pre-test post-test design to evaluate the effect of horticulture therapy on sleep quality among elderly residents of geriatric homes.

## Population and sample:

The target population consisted of elderly individuals residing in geriatric homes. A total of 50 participants were recruited through purposive sampling. Inclusion criteria were: age 60 years and above, presence of self-reported sleep problems, willingness to participate and ability to communicate. Exclusion criteria included severe psychiatric or neurodegenerative illness, current use of sedatives and critical illness preventing participation.

## Intervention - horticulture therapy:

Participants received structured horticulture therapy sessions for six weeks, five days a week, lasting 45-60 minutes each. Activities included preparing soil, sowing seeds, watering plants, removing weeds and observing plant growth. These tasks were designed to provide physical activity, sensory stimulation, relaxation and emotional satisfaction, thereby supporting better sleep. Sessions were facilitated and supervised to ensure consistency and safety.

## Tools for data collection:

The Pittsburgh Sleep Quality Index (PSQI) was used as the standardized tool for assessing sleep quality. It measures subjective sleep quality, sleep latency, sleep duration, sleep efficiency, disturbances, use of medication and daytime dysfunction. A structured demographic proforma was also administered to collect baseline characteristics such as age, gender, marital status and duration of stay.

## Data collection procedure:

In the pre-test phase, baseline sleep quality was measured using the PSQI before intervention. The participants then underwent six weeks of horticulture therapy. After the completion of sessions, the post-test assessment was conducted using the same tool to evaluate changes in sleep quality.

## Data analysis:

Data were organized and analyzed using descriptive and inferential statistics. Demographic data were summarized using frequency and percentage distribution. A paired t-test was applied to compare pre-test and post-test sleep quality scores, with a p-value < 0.05 considered statistically significant.

## Results:

Table 1 (see PDF) shows that most participants were aged 65-75 years (66%), male (54%) and Hindu (80%). A majority were separated/widowed (66%), urban residents (74%) and dependent mainly on "others" (40%) as their support system. Most had been staying for more than 2 years, were independent in daily activities, admitted involuntarily by children and had up to primary or secondary education. Table 2 (see PDF) show that mean score of sleep quality decreased from 2.92 (pre-test) to 1.18 (post-test) after horticulture therapy, with a mean difference of 1.74. The t-value of 27.768 and p < 0.001 indicate a highly significant improvement in sleep quality following horticulture therapy. Figure 1 (see PDF) show before intervention, the majority of participants (92%) had severe sleep problems, while none reported mild sleep problems. After horticulture therapy, 82% shifted to mild sleep problems, 18% to moderate problems and none remained in the severe category, indicating a significant improvement.

## Discussion:

The present study examined the effects of horticultural therapy (HT) on sleep quality among elderly individuals residing in geriatric homes and the findings demonstrated a statistically significant improvement in sleep quality following the six-week HT intervention. These results reinforce a growing body of literature supporting the role of nature-based therapies in promoting well-being among older adults. Multiple mechanisms may underlie the observed improvements. One is the psychological impact of HT, which includes stress reduction, mood enhancement and improved emotional regulation. Several studies have found that gardening reduces cortisol levels and enhances relaxation, which are conducive to better sleep [10, 11]. Additionally, HT fosters low-impact physical activity, which plays a critical role in modulating circadian rhythms and reducing sleep latency. This is consistent with prior findings that HT improved flexibility, lowered stress hormones and supported improved physical outcomes [11, 12]. HT also offers rich sensory engagement, which may promote neural relaxation and sleep. Activities involving touching soil, smelling plants and observing greenery activate sensory pathways linked to emotional regulation and parasympathetic activation [10]. Furthermore, HT provides purposeful engagement and social interaction, both of which are known to improve mood and sleep quality among older adults facing loneliness or depression [11, 13 and 14]. Notably, HT also produced biological improvements, as evidenced in previous studies that found increases in salivary IgA, α-amylase and chromogranin A, suggesting a beneficial immunomodulatory effect that may also support better sleep [10]. When compared with other non-pharmacological interventions-such as music therapy [15, 16-17], laughter therapy [18], warm water foot baths [19- 20] and light therapy [21]. HT offers a unique combination of emotional, physical, sensory and social benefits. While each therapy showed promise, HT may be more comprehensive in simultaneously addressing multiple factors affecting sleep.

This study also complements systematic reviews and meta-analyses that underscore the potential of HT as a holistic intervention for aging populations. Meta-analytic findings revealed consistent effects on reducing cortisol, promoting social interaction and enhancing quality of life [22-23]. While this study's findings are promising, certain limitations must be acknowledged. In the present study, chi-square analysis revealed no significant association between socio-demographic characteristics and baseline levels of stress, depression, or sleep quality, suggesting that the therapeutic effects of HT were independent of participants' age, gender, marital status, or education. This pattern is echoed in a randomized HT program by Han, Park and Ahn (2018), where improvements in stress reduction and physical functioning were observed irrespective of participants' mental health backgrounds [24]. Similarly, a 2022 study by Shen *et al.* found that a six-week HT intervention significantly improved sleep quality and mucosal immunity among elderly individuals across a wide age range (70-93), with no reported variation in outcomes based on personal characteristics [10]. Expanding on this, a meta-analysis by Yun *et al.* (2023) reviewed 32 HT studies and found consistent physical and psychological benefits-including stress reduction, enhanced flexibility and increased fruit and vegetable intake-across elderly populations, regardless of socio-demographic variation [11]. In Malaysia, Sham *et al.* (2020) implemented an edible gardening HT model and similarly reported improvements in cognitive and emotional health among elderly participants of differing ages, urban/rural backgrounds and health statuses [12]. Additionally, the study did not account for dietary, medication, or pre-existing mental health variables that may influence sleep quality. Future research should consider randomized controlled designs, larger sample sizes and longer follow-up periods to validate these results. This study adds to a growing body of literature supporting horticultural therapy as a low-risk, cost-effective and holistic intervention for improving sleep quality among institutionalized elderly individuals. The integration of HT into geriatric care settings may not only enhance sleep but also improve overall well-being and reduce dependence on pharmacological sleep aids.
